# Withaferin A Rescues Brain Network Dysfunction and Cognitive Deficits in a Mouse Model of Alzheimer’s Disease

**DOI:** 10.3390/ph18060816

**Published:** 2025-05-29

**Authors:** Linhan Yang, Yang Zou, Jihua Fan, Pu Yin, Han Qin, Zhen Li, Fengjuan Wu, Xingyi Li, Huaijin Teng, Yun Zhang, Xiaowei Chen, Sunny C. Li

**Affiliations:** 1 Guangxi Key Laboratory of Special Biomedicine/Advanced Institute for Brain and Intelligence, School of Medicine, Guangxi University, Nanning 530004, China; ylh200423@163.com (L.Y.); zy13878131507@163.com (Y.Z.); fanjihua32@163.com (J.F.); 2207301169@st.gxu.edu.cn (P.Y.); xiaowei_chen@tmmu.edu.cn (X.C.); 2 LFC Laboratory and Chongqing Institute for Brain and Intelligence, Guangyang Bay Laboratory, Chongqing 400064, China; hanqin@cibi.ac.cn (H.Q.); teng301@sina.cn (H.T.); 3 College of Intelligence Science and Technology, National University of Defense Technology, Changsha 410073, China; lizhen20@nudt.edu.cn; 4 Department of Neurology, Lanzhou University, Lanzhou 730000, China; wfj5678@outlook.com; 5 Center for Neurointelligence, School of Medicine, Chongqing University, Chongqing 400044, China; xingyi_li@cqu.edu.cn; 6 Brainspace Research Unit, Chongqing 400064, China; 7 State Key Laboratory of Structural Chemistry, Fujian Institute of Research on the Structure of Matter, Chinese Academy of Sciences, Fuzhou 350002, China; 8 Brain Research Center and State Key Laboratory of Trauma and Chemical Poisoning, Third Military Medical University, Chongqing 400038, China; 9 NewLight Neuroscience Unit, Chongqing 400064, China

**Keywords:** Alzheimer’s, withaferin A, cortical slow-wave activity, cognitive function, neuroprotection

## Abstract

**Background:** Alzheimer’s disease (AD) is the most common dementia, characterized by significant cognitive impairments and neural network dysfunction. Currently, multiple therapeutic strategies are being developed to design effective anti-AD drugs. Among them, Withaferin A (WA), a natural steroidal lactone extracted from Withania somnifera leaves, has been shown to reduce amyloid-β (Aβ) peptide levels in vitro. However, its potential to improve cognitive function in AD remains unclear. **Methods:** In this study, 5xFAD mice were administered WA (2 mg/kg intraperitoneally every 2 days) for 14 days, and its neuroprotective effects were evaluated through behavioral tests, wide-field imaging, immunohistochemistry, and ELISA. **Results:** WA significantly improved short-term memory, as evidenced by enhanced performance in the Novel Object Recognition Test (NORT) (*p* < 0.001, *n* = 10), Novel Location Recognition Test (NLRT) (*p* < 0.01, *n* = 14), and Three-Chamber Social Test (TCST) (*p* < 0.001, *n* = 8). WA also ameliorated long-term memory deficits in the Morris Water Maze Test (MWMT) (*p* < 0.05, *n* = 7). Furthermore, cortical wide-field Ca^2+^ imaging revealed that WA treatment rescued slow-wave impairments by enhancing long-range coherence (0.8363 ± 0.0185, *p* < 0.01, *n* = 8) and reducing the frequency of slow-wave activity (0.6578 ± 0.0512 Hz, *p* < 0.01, *n* = 8). Additionally, WA treatment significantly reduced Aβ plaque deposition in both cortical and hippocampal regions. **Conclusions:** These findings suggest that WA may be a promising therapeutic agent for AD, exerting neuroprotective effects.

## 1. Introduction

Alzheimer’s disease (AD) is the most common form of dementia in the elderly [1,2], characterized by progressive impairments in memory and cognition. Over the past two decades, most drug development efforts have focused on targeting Aβ peptide antibodies [3,4]. Among them, donanemab and lecanemab have exhibited adequate removal of Aβ plaques from patients diagnosed with AD [5,6]. Nevertheless, clinical trials indicate these treatments showed limited improvement in cognitive function [7].

Several hypotheses have been proposed to explain the pathogenesis of AD, including excessive Aβ accumulation, tau hyperphosphorylation, neuroinflammation, synaptic dysfunction, oxidative stress [8], neurotrophic deficits, and neuronal loss [9,10,11]. Recent studies indicate that neuronal hyperactivity contributes to brain network dysfunction and cognitive impairment in AD [12]. Wide-field imaging to monitor Ca^2+^ fluorescent signals reveals impaired propagation of cortical slow wave activities (0.1–3 Hz), i.e., low-frequency, high-amplitude oscillations, in anesthetized AD mouse models [13,14]. Importantly, restoring normal slow-wave activity has been associated with improvements in cognitive function [15]. Therefore, identifying novel therapeutic agents that can restore neuronal network function may be key to improving cognitive outcomes in AD.

Emerging evidence demonstrates that diverse natural and synthetic antioxidant compounds exhibit therapeutic potential in AD management through mechanisms including redox homeostasis modulation and neural circuit protection [8]. Withaferin A (WA) is a natural steroidal lactone derived from *Withania somnifera* [16], known for its anti-inflammatory, antioxidant, anti-tumor, and immunomodulatory properties [1,15,17,18]. Pharmacokinetic studies have demonstrated WA’s high safety profile (as evidenced by a Phase I clinical trial) [17,19], its short half-life [20,21], and its ability to cross the blood–brain barrier [22]. While WA has been shown to reduce Aβ production in vitro [23], its effects on neuronal network function and cognition in in vivo AD models remain unclear.

Therefore, a combination approach targeting neuroprotection and cognitive function is essential. Both in vivo and in vitro experiments demonstrated that WA can significantly reduce the levels of several neuroinflammatory factors (such as TNF-α, IL-6, etc.) in AD mice, suggesting its neuroprotective effects [24,25,26]. Given that WA has been shown to reduce Aβ production in vitro [23], we hypothesized that WA can rescue the deficits in neuronal network function and memory in AD mice. Here, we demonstrated that a 14-day WA treatment regimen (2 mg/kg i.p. every 2 days) significantly improved short-term memory (as assessed via NORT, NLRT, and TCST) and long-term memory (MWMT) in 5xFAD mice. Furthermore, cortical wide-field Ca^2+^ imaging revealed that WA treatment restored slow-wave activity by enhancing long-range coherence and reducing slow-wave frequency. Finally, immunostaining and enzyme-linked immunosorbent assay (ELISA) analysis of WA-treated 5xFAD mice showed that Aβ levels were reduced. These findings suggest that WA holds promise as a potential therapeutic agent for AD.

## 2. Results

### 2.1. WA Improves Cognitive Deficits in 5xFAD Mice

**Figure 1:**
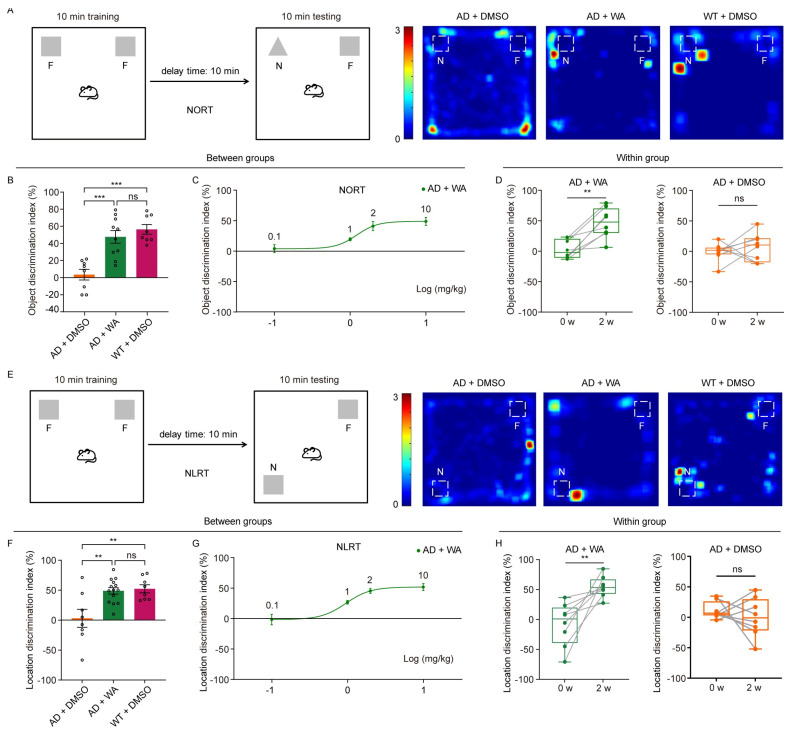
WA improves recognition memory in 5xFAD mice. (**A**) Experimental protocol for the NORT and representative heatmaps showing time distribution around each object. (**B**) Object discrimination index at 2 weeks. 5xFAD mice were treated with WA (*n* = 10) or DMSO (*n* = 8), and WT mice were treated with DMSO (*n* = 8). N, novel; F, familiar. (**C**) Summary change curve of the object discrimination index across varying WA doses (0.1 mg/kg, *n* = 8; 1 mg/kg, *n* = 8; 2 mg/kg, *n* = 8; and 10 mg/kg, *n* = 8). (**D**) Object discrimination index before and after WA treatment during the testing phase. 5xFAD mice were treated with WA (*n* = 8) or DMSO (*n* = 8). (**E**) Experimental protocol for the NLRT and representative heatmaps showing time distribution around each object. N, novel; F, familiar. (**F**) Location discrimination index at 2 weeks. 5xFAD mice were treated with WA (*n* = 14) or DMSO (*n* = 8), and WT mice were treated with DMSO (*n* = 8). (**G**) Summary change curve of the location discrimination index across varying WA doses (0.1 mg/kg, *n* = 8; 1 mg/kg, *n* = 8; 2 mg/kg, *n* = 8; and 10 mg/kg, *n* = 7). (**H**) Location discrimination index before and after WA treatment during the test phase. 5xFAD mice were treated with WA (*n* = 8) or DMSO (*n* = 8). Each dot represents an individual animal. Data in (**B**,**C**,**F**,**G**) are presented as the mean ± SEM. Data in (**B**,**F**) are analyzed using one-way ANOVA. Data in (**D**,**H**) are analyzed using a paired *t*-test. n.s., *p* > 0.05; **, *p* < 0.01; and ***, *p* < 0.001.

**Figure 2:**
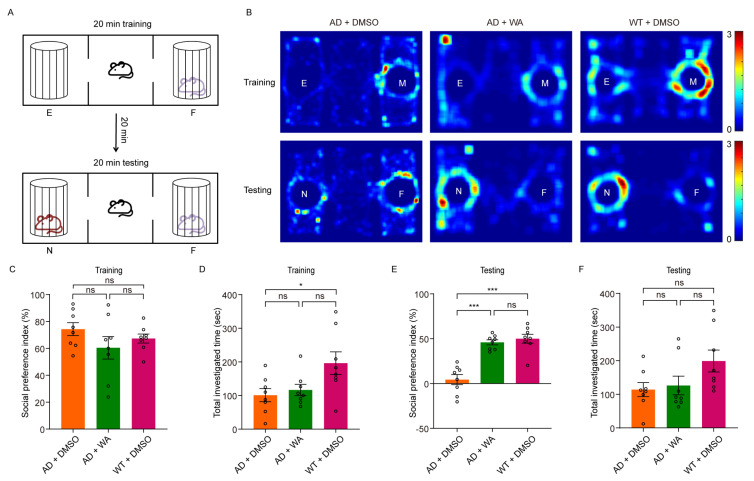
WA improves social memory in 5xFAD mice. (**A**) Experimental protocol for the Three-Chamber Social Test (TCST). E, empty; M, mouse; N, novel; F, familiar. (**B**) Representative heatmaps showing time distribution in the TCST. (**C**,**E**) Social preference index during training (**C**) and testing (**E**) in the social novelty task. (**D**,**F**) Total investigation time during training (**D**) and testing (**F**) in the social novelty task. 5xFAD mice were treated with WA (*n* = 8) or DMSO (*n* = 8), and WT mice were treated with DMSO (*n* = 8). Each dot represents an individual animal. Data in **C**–**F** are presented as the mean ± SEM. Data in (**C**–**F**) are analyzed using one-way ANOVA. n.s., *p* > 0.05; *, *p* < 0.05; and ***, *p* < 0.001.

**Figure 3:**
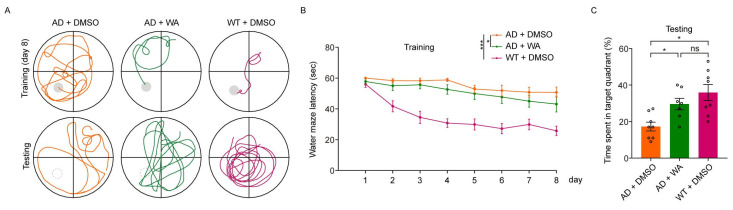
WA improves long-term memory in 5xFAD mice via the Morris Water Maze Test (MWMT). (**A**) Representative swim path tracings from the MWMT. The grey solid circles represent the platform, and the grey dashed circles represent the removal of the platform. (**B**) Escape latency during the training phase of the MWMT. (**C**) Time spent in the target quadrant during the probe test. 5xFAD mice were treated with WA (*n* = 7) or DMSO (*n* = 8), and WT mice were treated with DMSO (*n* = 8). Each dot represents an individual animal. Data in (**B**,**C**) are presented as the mean ± SEM. Data in (**B**) are analyzed using two-way ANOVA. Data in (**C**) are analyzed using one-way ANOVA. n.s., *p* > 0.05; *, *p* < 0.05; and ***, *p* < 0.001.

- To assess whether chronic WA treatment improves cognitive function in 5xFAD mice, we conducted a series of behavioral tests in transgenic AD amyloidosis mouse models. Female 5xFAD mice were treated with WA (2 mg/kg every 2 days) or 1% (*v*/*v*) dimethyl sulfoxide (DMSO) as control for 14 days.
- To assess the effect of WA on short-term memory in 5xFAD mice, we first performed the Novel Object Recognition Test (NORT) and Novel Location Recognition Test (NLRT) (Figure 1). The mice were placed in an open-field box with two identical objects and allowed to explore freely for 10 min (Figure 1A,E). After a 10 min delay, the same mice were reintroduced to the box, where one object was replaced with a novel object (NORT) or relocated to a new location (NLRT), and exploration was recorded for another 10 min. The discrimination index, calculated as the difference in time spent exploring novel versus familiar objects (NORT) or locations (NLRT), was used to assess cognitive function.
- Compared to age-matched AD control and wild-type (WT) mice, WA-treated mice exhibited significantly improved performance in both NORT and NLRT, as indicated by an increased discrimination index for novel objects (Figure 1B,C) and novel locations (Figure 1F,G). In contrast, AD control mice showed no significant improvement (Figure 1D,H).
- To evaluate the dose-dependent effects of WA, we tested multiple doses (0.1, 1, 2, and 10 mg/kg) in 5xFAD mice (Figure 1C,G). While lower doses (0.1–1 mg/kg) had no significant impact on non-spatial or spatial memory, higher doses (2 and 10 mg/kg) significantly enhanced cognitive performance, suggesting a dose-dependent effect of WA on cognitive function.
- Next, we assessed social memory using the Three-Chamber Social Test (TCST) after 14 days of WA treatment. During the training phase, mice were placed in a chamber with one compartment containing a stimulus mouse and another empty compartment, allowing free exploration for 20 min (Figure 2A,B). No differences were observed in social preference or investigation time between the WA-treated and AD control groups during this phase (Figure 2C,D). However, WA-treated mice demonstrated a significant preference for the chamber containing an unfamiliar mouse during the testing phase (Figure 2E). Despite this, no significant difference was observed in the overall investigation time (Figure 2F).
- To assess long-term memory, we conducted the Morris Water Maze Test (MWMT). As shown in Figure 3B, WA treatment ameliorated learning deficits in 5xFAD mice, with a notable reduction in escape latency compared to AD control mice. In the probe test on day 9, WA-treated mice crossed the target quadrant more frequently and spent more time in the target quadrant than AD control mice (Figure 3A,C), indicating improved spatial memory function.

### 2.2. WA Treatment Improves Long-Range Coherence of Slow-Wave Activity

To evaluate the effect of WA on cortical network dynamics, we used wide-field Ca^2+^ imaging to quantify neuronal activity patterns in anesthetized AD mice (Figure S1A). A large craniotomy was performed to expose one hemisphere (Figure S1B), followed by bulk cortical loading with the calcium indicator Cal-520 AM. Low-dose isoflurane anesthesia (0.8–1.0%) induced characteristic slow-wave oscillations (<1 Hz), resembling those observed during natural NREM sleep [13]. The recorded Ca^2+^ signals reflected an aggregate of neuronal and neuropil activity [27].

Cortical slow-wave activities were mapped across the hemisphere as previously described [28]. Shortly, the cortex was divided into four regions of interest: frontal, motor, somatosensory, and occipital regions (Figure S1B) [14]. Synchronization dynamics of these regions were quantified through cross-regional coherence analysis, with high coherence values (close to 1) indicating synchronized activity and low coherence values (close to 0) indicating desynchronized activity.

**Figure 4:**
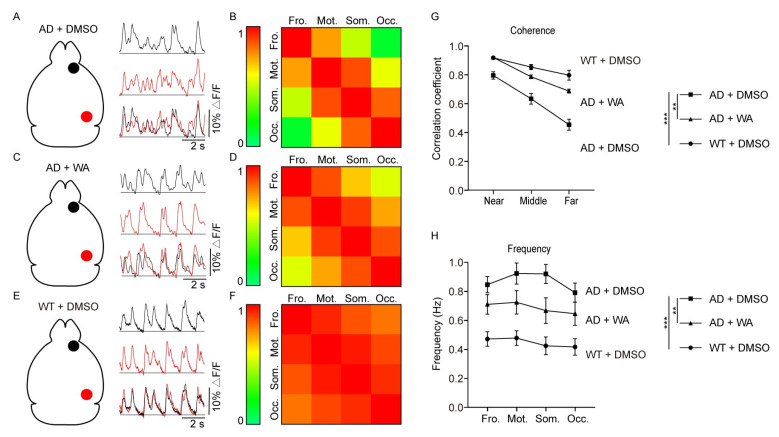
WA improves long-range coherence of slow-wave activity in 5xFAD mice. (**A**,**C**,**E**) Schematic diagrams of the injection sites and comparison of slow waves in the AD control group (**A**), the treatment group (**C**), and the WT control group (**E**). Black circles and lines indicate the frontal cortical regions and their associated slow-wave activity, whereas red circles and lines correspond to the occipital cortical regions and their respective slow-wave activity. (**B**,**D**,**F**) Cross-correlation matrices from three representative experiments created from the cortical domains in the AD control group (**B**), the treatment group (**D**), and the WT control group (**F**). (**G**) Summary plot displaying the average cross-correlation coefficients and standard errors plotted against cortical distance. (**H**) Summary plot of the mean frequencies of slow waves along the anteroposterior cortical axis. 5xFAD mice were treated with WA (*n* = 8) or DMSO (*n* = 8), and WT mice were treated with DMSO (*n* = 8). Data in G and H are presented as the mean ± SEM and analyzed using two-way ANOVA. **, *p* < 0.01; and ***, *p* < 0.001.

In WT mice, slow-wave activity demonstrated pan-cortical uniformity (pan-cortical uniformity refers to the temporal synchronization of neuronal activities across the entire cerebral cortex) in Ca^2+^ transients, with synchronized traces observed between regions (Figure 4E,F). In contrast, 5xFAD mice displayed disorganized Ca^2+^ signal patterns, reflecting desynchronization of cortical activities (Figure 4A,B). Notably, this disruption was significantly restored in 5xFAD mice treated with WA (Figure 4C,D), as the spatiotemporal pattern of slow-wave activities returned regularity across the cortex, resembling the patterns observed in DMSO-treated WT mice (Figure 4E,F).

To further quantify coherence differences between groups, we analyzed the correlation of Ca^2+^ signals as a function of distance between cortical regions, categorized as adjacent (near), intermediate (middle; one intervening region), or distal (far; two regions between, e.g., occipital-frontal) [14]. WT mice exhibited sustained high coherence, exhibiting minimal distance-dependent attenuation. However, in 5xFAD control mice, coherence was significantly reduced, particularly between distant cortical regions. In contrast, WA-treated 5xFAD mice exhibited markedly higher coherence across all distances compared to 5xFAD control mice. A two-way analysis of variance (ANOVA) demonstrated significant differences across experimental groups (Figure 4G). Similarly, the slow-wave frequency in different cortical regions of WA-treated 5xFAD mice more closely resembled that of DMSO-treated WT mice than DMSO-treated 5xFAD mice (Figure 4H), indicating that 14-day WA treatment ameliorated both AD-associated desynchronization and increased the frequency of cortical slow waves in 5xFAD mice.

### 2.3. WA Reduces Aβ Deposition in 5xFAD Mice

**Figure 5:**
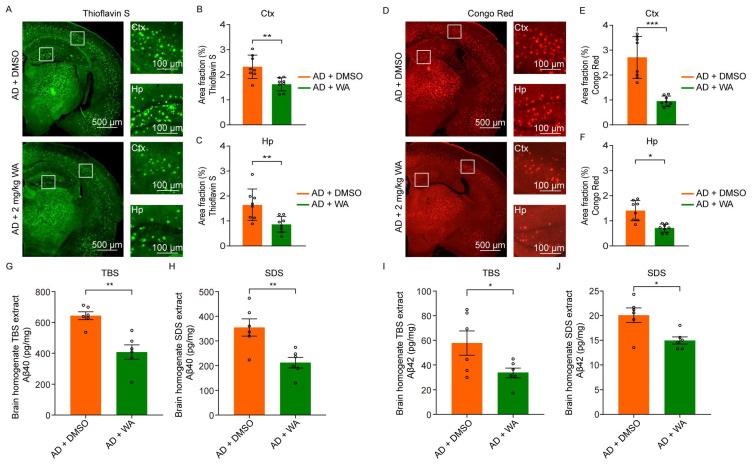
WA ameliorates Aβ deposition in 5xFAD mice. (**A**,**D**) Thioflavin S staining (**A**) and Congo Red staining (**D**) of the WA treatment group and AD control group. The inset shows a magnified view of a representative image in the cortex (Ctx) and the hippocampus (Hp). (**B**,**C**) Comparison of Thioflavin S -positive plaques in the cortex (Ctx) (**B**) and hippocampus (Hp) (**C**) of the WA treatment group (*n* = 8) and AD control group (*n* = 8). (**E**,**F**) Comparison of Congo Red-positive plaques in the cortex (Ctx) (**E**) and hippocampus (Hp) (**F**) of the WA treatment group (*n* = 8) and AD control group (*n* = 8). (**G**,**H**) Quantification of Aβ_40_ using ELISA in TBS (**G**) and SDS (**H**) of brain homogenates of the WA treatment group (*n* = 6) and AD control group (*n* = 6). (**I**,**J**) Quantification of Aβ_42_ using ELISA in TBS (**I**) and SDS (**J**) of brain homogenates of the WA treatment group (*n* = 6) and AD control group (*n* = 6). Each dot represents an individual animal. Data in (**B**,**C**,**E**–**J**) are presented as the mean ± SEM and analyzed using an unpaired *t*-test. *, *p* < 0.05; **, *p* < 0.01; and ***, *p* < 0.001.

Given that Aβ plaques are the pathological hallmark of AD, we evaluated Aβ deposition in 5xFAD mice using thioflavin S staining (Figure 5A). WA-treated 5xFAD mice exhibited a significant reduction in Aβ plaque burden in the cortex and hippocampus compared to the vehicle-treated AD group (Figure 5B,C). Consistently, Congo Red staining (Figure 5D) further confirmed that Aβ plaque deposition in WA-treated AD mice was also reduced relative to AD controls (Figure 5E,F).

We also quantified the soluble and insoluble Aβ levels in the cortex of 5xFAD mice using ELISA. Compared to untreated AD controls, 14-day WA administration significantly reduced soluble Aβ_40_ and Aβ_42_ levels in tris-buffered saline (TBS) extracts (Figure 5G,I). A corresponding decrease in insoluble Aβ_40_ and Aβ_42_ was also detected in sodium dodecyl sulfate (SDS)-extracted fractions (Figure 5H,J).

## 3. Discussion

Cognitive dysfunction in AD primarily results from severe impairments in neural network function [29], neuroinflammation [30], and Aβ deposition in the brain [31]. WA has been shown to reduce the aggregation of Aβ in vitro [23], suggesting its potential for AD treatment. In this study, WA treatment for 14 days ameliorated cognitive dysfunction in 5xFAD mice, as demonstrated by short-term and long-term memory tests. Additionally, WA enhanced slow-wave activity coherence, as observed through wide-field Ca^2+^ imaging. Furthermore, WA demonstrated therapeutic effects by significantly reducing Aβ plaque deposition in the AD animal model, consistent with a previous study that showed WA can reduce Aβ production in vitro [23]. These findings suggest that WA treatment demonstrates neuroprotective potential.

Therapeutic efficacy is evaluated through cognitive function improvement in AD [32]. Our study mainly validated this paradigm, with WA demonstrating comparable improvement to donepezil in behavioral assays, including the NORT and MWMT [33].

In the early stages of AD, abnormal neuronal hyperactivity precedes Aβ plaque aggregation and is correlated with cognitive impairment [34,35]. Excessive glutamate leads to sustained neuronal hyperactivity [36], resulting in synaptic integrity loss and impaired plasticity [35]. This hyperactivity may extend beyond local neuronal interactions, affecting long-range brain networks. Wide-field Ca^2+^ imaging studies have shown that cortical slow-wave oscillations are disrupted in anesthetized transgenic AD model mice [14]. Consistent with this hypothesis, our wide-field imaging revealed diminished slow-wave activity coherence (Figure 4G) across cortical regions in AD mice, attributable to neuronal hyperexcitability concomitant with elevated slow-wave frequency (Figure 4H). The above results indicate that WA treatment improves neural network function. Previous studies have shown that the presence of soluble Aβ may interfere with synaptic transmission between neurons, which could disrupt the balance between excitatory and inhibitory neurons, thereby leading to abnormal neuronal network activity [37]. Consistent with this notion, we found that both soluble and insoluble Aβ levels in brain homogenates were significantly reduced after 14 days of WA treatment (Figure 5). Slow waves, which are prominent during non-rapid eye movement (non-REM) sleep [14], are crucial for memory consolidation [14,38], metabolic waste clearance [12], and neuronal plasticity [39]. Furthermore, studies in mice and humans have demonstrated that suppressing neuronal hyperactivity can improve or even restore cognitive function [40,41]. Notably, WA treatment appears to improve cognitive function and restore long-range slow-wave synchrony through neuroprotective mechanisms.

The mechanism underlying WA’s therapeutic effects remains under investigation. WA may exert its neuroprotective effects through the mechanism of inhibiting neuroinflammation. WA has been proposed to counteract neuroinflammatory damage in various disease models, including those involving both motor and cognitive dysfunction [42,43]. In AD models, Aβ promotes neuroinflammation and reactive oxygen species (ROS) production [8,44], leading to excitatory–inhibitory neurotransmitter imbalance and neuronal hyperactivity [45]. WA may mitigate this pathology via the NF-κB pathways [1,26,46,47]. NF-κB activation promotes nuclear translocation and inflammatory cytokine expression, disrupting neuronal communication [48,49] and impairing cognitive function [50]. WA has been shown to decrease pro-inflammatory cytokines (IL-6, IL-1β, and TNF-α) [17,51,52] while increasing anti-inflammatory cytokines (TGF-β1, IL-10, IL-4, and IL-13) [51,53,54]. Additionally, WA improves microglial and astrocytic functions, thereby protecting neurons and glial cells from neuroinflammation and restoring neural network integrity [50]. Furthermore, neuroinflammation suppression may attenuate the production and deposition of Aβ [55,56]. This hypothesis suggests potential involvement of distinct Aβ clearance mechanisms, such as microglial phagocytosis of Aβ oligomers [57], although further mechanistic studies are warranted.

While the precise cellular and molecular mechanisms of the neuroprotective effect of WA in AD mice remain unknown, it showed similar improvements in cognitive functions as United States Food and Drug Administration (US-FDA)-approved AD drugs, including donepezil (an acetylcholinesterase inhibitor) and memantine (an NMDA receptor antagonist) [58,59,60,61]. Mechanistically, these agents may differ from each other, as each could specifically target different cell signaling cascades for neural network function and AD-related pathology. The current study acknowledges limitations in direct pharmacological comparisons between WA and reference drugs regarding their complete therapeutic profiles. Future investigations should establish quantitative benchmarks against existing therapies and explore WA-containing combination regimens targeting complementary AD pathways to potentiate treatment efficacy.

Despite its broad pharmacological profile, the application of WA as an ideal therapeutic agent is limited by its poor aqueous solubility. Our study also observed this issue with WA. Moreover, interspecies differences in blood–brain barrier (BBB) permeability between mice and humans may reduce the bioavailability of WA in clinical settings [62,63,64], even though it can cross the BBB in mice [22]. To overcome these limitations, several strategies have been proposed to enhance its efficacy, including nanoparticle formulation [65], structural modification [17], and combination therapy with multiple agents [66]. These approaches not only improve the bioactivity of WA but also expand its therapeutic potential against various diseases, including AD.

In summary, WA significantly improves cognitive and neuronal network functions via neuroprotection in adult female 5xFAD mice. The multifactorial nature of AD pathogenesis limits the efficacy of currently approved treatments, which primarily include symptomatic and disease-modifying drugs. While anti-Aβ antibodies have emerged as a primary therapeutic strategy, their cognitive benefits remain limited [7]. Therefore, a combination approach targeting neuroprotection and cognitive preservation is essential [67]. This study highlights the neuroprotective properties of WA, which alleviates neural network dysfunction and cognitive impairments. These findings underscore WA’s potential as a promising therapeutic candidate for AD and a valuable avenue for future drug development.

## 4. Materials and Methods

### 4.1. Animals

All experimental procedures were conducted in accordance with the National Institutes of Health Guide for the Care and Use of Laboratory Animals and the animal use protocol that was reviewed and approved by the Institutional Animal Use and Care Committee at Guangxi University (Gxu-2025-l85, 2024.06.10). The experiments were performed using 6–7-month-old 5xFAD mice (Tg6799, C57BL/6 background), which carry mutations in human APP (KM670/671NL, I716V, and V717I) and PSEN1 (M146L and L286V). Control groups consisted of age-matched WT littermates.

Female 5xFAD transgenic mice and their wild-type (WT) counterparts were sourced from Jiangsu Jinzhihe Biotechnology Co., Ltd. (Yangzhou, China). The mice were kept in a controlled vivarium setting with maintained ambient temperature (20–23 °C) and humidity (50–60%) and were maintained under a 12 h light/dark cycle. Standard rodent chow and filtered water were available without restriction.

The exclusive use of female 5xFAD mice was based on two key factors: (1) the well-documented female predominance in AD progression, particularly the accelerated Aβ deposition in postmenopausal pathophysiology [68], and (2) the established gender-specific neuropathological features in 5xFAD mice, including earlier cortical Aβ plaque formation and more severe hippocampal synaptic degeneration [69]. This gender-stratified approach enhances the sensitivity to detect therapeutic effects within the 14-day study period and reflects the epidemiological risk profile of AD.

### 4.2. WA Treatment

WA (#W864480-25mg, YUANYE, Shanghai, China) was initially dissolved in 100% dimethyl sulfoxide (DMSO) to prepare a 100 mg/mL stock solution, which was subsequently diluted with sterile phosphate-buffered saline (PBS) to achieve a final DMSO concentration of 1% (*v*/*v*) for intraperitoneal (i.p.) administration. The control groups received i.p. injections of the same volume of 1% (*v*/*v*) DMSO diluted in PBS following identical treatment schedules. The mice were randomly divided into three experimental cohorts: the treatment group (AD + 2W) received intraperitoneal injections of 1 μg/μL WA at a volume of 0.05 mL (2 mg/kg) every two days; the AD control group (AD + DMSO) and the WT control group (WT + DMSO) both received intraperitoneal injections of 0.05 mL 1% (*v*/*v*) DMSO every two days.

Except for the dose-dependent testing shown in Figure 1C,G, all treatment groups received 2 mg/kg WA. The selected dose (2 mg/kg) was based on previously published studies [20,25]. In addition, we conducted systematic dose–response analyses (0.1–10 mg/kg), showing maximal cognitive efficacy at this dose, as evidenced by plateaued discrimination indices in behavioral tests (NORT and NLRT) without further improvement at higher doses (Figure 1).

### 4.3. Novel Object Recognition/Novel Location Recognition Test (NORT/NLRT)

The NORT/NLRT task consisted of three phases: habituation, training, and testing [70]. First, 24 h prior to testing, animals were acclimated to the experimental arena (identical to that used in the open field test) during a 10 min unrestricted exploration session. During training, two identical objects were placed 5 cm from the adjacent walls on either side of the arena. Each mouse was positioned in the center, facing away from the objects, and allowed to explore for 10 min before being returned to its home cage. Testing occurred 10 min later. In the NORT testing phase, one of the objects was replaced with a new object of a different shape but placed in the same position. In the NLRT testing phase, one object’s position was swapped to the opposite side of the arena. The mice were again allowed to explore for 10 min. All behavioral sessions were video-recorded for subsequent analysis, with subject positioning quantified using MATLAB-based tracking algorithms (custom script). The apparatus was sanitized with 75% ethanol solution between trials.

Discrimination index = (T_novel_ − T_familiar_)/(T_novel_ + T_familiar_) × 100% (1)

### 4.4. Three-Chamber Social Test (TCST)

The Three-Chamber Social Test (TCST) was used to assess social novelty preference and social abilities [71]. The behavioral arena consisted of three identical compartments (25 × 50 × 40 cm each) created using transparent acrylic partitions within a rectangular enclosure (75 × 50 × 40 cm). Each side chamber contained a cylindrical cage (diameter: 10 cm, height: 15 cm), allowing for visual and olfactory interactions without direct contact. Mice were individually housed for seven days prior to the test. The experiment consisted of three phases: habituation, training, and testing. The habituation phase (Day 1) consisted of a 20 min unrestricted exploration period. During the subsequent test day (Day 2), two wire cages were positioned in opposing chambers: one empty (control) and one containing a sex-matched unfamiliar conspecific (Stranger Mouse 1). The experimental mouse was placed in the central chamber and allowed to explore for 20 min, followed by a 20 min rest period in its home cage. During the testing phase, another novel mouse of the same sex (Stranger Mouse 2) was placed in the previously empty cage, and the experimental mouse was again allowed to explore for 20 min. All behavioral sessions were video-recorded for subsequent analysis, with subject positioning quantified using MATLAB-based tracking algorithms (custom script). The apparatus was sanitized with 75% ethanol solution between trials.

Social preference index = ((time for novel Stranger Mouse 2) − (time for familiar Stranger Mouse 1))/((time for novel Stranger Mouse 2) + (time for familiar Stranger Mouse 1)) × 100%(2)

### 4.5. Morris Water Maze Test (MWMT)

The Morris Water Maze Test (MWMT) was used to measure hippocampus-dependent spatial memory [72]. The test was performed in a circular water maze (120 cm diameter × 40 cm height) containing temperature-controlled water (20 ± 1 °C) opacified with titanium dioxide (1 g/L). Four imaginary quadrants were established, with a submerged escape platform (10 cm diameter × 10 cm height) positioned 30 cm from the perimeter and maintained 1 cm below the water surface throughout acquisition trials. Distinct visual cues were mounted on the surrounding walls to facilitate spatial orientation. Training began after one day of habituation and continued for eight days (three training trials per day, separated by 15 min, starting at the same time each day for eight days). During each trial, the mice were released from random starting positions and allowed up to 60 s to locate the platform, remaining on it for 30 s after finding it. If the platform was not located within 60 s, mice were guided to the platform and allowed to stay for 30 s. On the ninth day, the platform was removed, and a test was conducted, allowing mice to swim freely for one minute to search for the platform. During all training and test trials, videos were recorded, and the animals’ locations were tracked using a custom MATLAB program. The following metrics were calculated: escape latency (s) and the percentage of time spent in the target quadrant (%).

### 4.6. Wide-Field Fluorescence Imaging

The experimental procedures were adapted from previously published reports [14]. Mice were anesthetized with 1–1.5% isoflurane in air and maintained with 0.8–1% isoflurane during surgical procedures. A cranial window was created on one cortical hemisphere while preserving the dura mater. The cortex was subdivided into four regions along the anteroposterior axis of each hemisphere, roughly corresponding to the frontal, motor, somatosensory, and occipital cortices. Using a microinjector (RWD, R-480), 1000 nL of Cal-520 AM was pressure-injected into the cortical layers at a depth of 0.55–0.6 mm in each of the four regions. This procedure labeled nearly the entire cortical extent.

Wide-field fluorescence imaging was conducted using the 2-Channel Alternating exposure wide-Field Explorer (2-CAFE) system (Chongqing NewLight Co., Ltd., Chongqing, China) with a high-speed digital camera (Ximea, MC124MG-SY, Beijing, China). Images were acquired at a frame rate of 35 fps (2048 × 2048 pixels).

### 4.7. Imaging Data Analysis

The experimental procedures were adapted from previously published reports [28]. Custom software packages, including LabVIEW 2014, Igor Pro 5.0 (Wavemetrics Inc., Portland, OR, USA), and MATLAB 2016b (MathWorks, Natick, MA, USA), were utilized for offline data analysis to ensure accuracy and reproducibility of the results. Calcium signaling was quantified using the LabVIEW-based LOTOS program, which calculated relative fluorescence changes (ΔF/F = (F − F₀)/F₀) for each region of interest (ROI), where F₀ was defined as the 25th percentile of baseline fluorescence intensity for that ROI. To address cardiac-related artifacts, which typically occur at frequencies above 4 Hz and may interfere with the signal of interest, a custom MATLAB script based on fast Fourier transform (FFT) was implemented to effectively filter out these noise components.

To evaluate the spatial coherence of cortical Ca^2+^ signals, each cortical hemisphere was anatomically divided into four anteroposterior regions: the frontal cortex, motor cortex, somatosensory cortex, and occipital cortex. Coherence between these cortical regions was assessed using the mean Ca^2+^ signal over a 30 s time window. The frequency of Ca^2+^ transients was determined through a two-step peak detection algorithm. Initially, the raw Ca^2+^ transient data were smoothed using a moving average window with a span of 3. Peaks detected in the first round were required to exhibit a minimum prominence equivalent to one standard deviation of the baseline signal, with a maximum of one peak per second allowed. In the subsequent refinement step, peak identification was further constrained by the average peak width, allowing for precise determination of the final peak components.

### 4.8. Thioflavin S Staining

The experimental procedures were adapted from previously published reports [28]. The presence of Aβ deposits was assessed using Thioflavin S fluorescence staining. Tissue sections were treated with 0.002% Thioflavin S solution (Sigma-Aldrich, Shanghai, China, Cat# T1892-25G) in 50% ethanol (10 min incubation) and sequentially washed (2 × 50% ethanol, 1 × PBS). Following fixation, tissue sections were mounted with antifade mounting medium and visualized under a laser scanning confocal microscope (OLYMPUS, Beijing, China, FV3000).

### 4.9. Congo Red Staining

The experimental procedures were adapted from previously published reports [28]. For detection of Aβ deposits, tissue sections were stained with Congo Red (Sigma-Aldrich, C6767, Shanghai, China). Sections were incubated in 0.01% Congo Red solution (75% ethanol vehicle) for 10 min and subjected to sequential washes (2 × 50% ethanol, 1 × PBS). Following fixation, tissue sections were mounted with antifade mounting medium and visualized under a laser scanning confocal microscope (OLYMPUS, Beijing, China, FV3000).

### 4.10. Aβ Enzyme-Linked Immunosorbent Assay (ELISA)

For biochemical assessments, mice were transcardially perfused with PBS prior to brain collection. Cerebral tissues were cryogenically homogenized using liquid nitrogen in a pre-chilled mortar. The homogenate was suspended in 0.1 M TBS (pH 7.4) and centrifuged (13,000× *g*, 4 °C, 1 h) to separate soluble Aβ fractions. The resulting pellet was resuspended in 2% SDS buffer and recentrifuged under identical conditions to extract insoluble Aβ species. Aβ_40_ and Aβ_42_ concentrations were determined using commercial ELISA kits (Elabscience, Wuhan, China: E-EL-M3009 for Aβ_40_, E-EL-M3010 for Aβ_42_) according to the manufacturer’s specifications, with the final values normalized to the total protein content.

### 4.11. Data Analysis and Statistics

Behavioral analyses were conducted under experimental conditions. For comparisons involving multiple groups, two-way ANOVA or one-way ANOVA was employed. For comparisons within the same group, a paired *t*-test was used. A *p*-value < 0.05 was considered statistically significant. Data are presented as the mean ± SEM. Statistical analyses were performed using GraphPad Prism (version 8.4.2, GraphPad Software, Inc., Boston, MA, USA).

## 5. Conclusions

In conclusion, WA improves neural network function through neuroprotective mechanisms, as evidenced by the enhancement of slow-wave activity synchrony and frequency and, ultimately, the restoration of memory loss in 5xFAD mice. WA may exert its neuroprotective effects through inhibiting neuroinflammation and the reduction of AD-related pathology. Furthermore, with further investigation into its comprehensive pharmacological profile and potential clinical applications, WA holds promise as a novel therapeutic candidate for the treatment of AD.

## Data Availability

The raw data that support the findings in this paper are available as source data and are included in this manuscript.

## Supplementary Materials

The following supporting information can be downloaded at https://www.mdpi.com/article/10.3390/ph18060816/s1: Figure S1: Schematic of wide-field imaging. (A) Schematic of cortical wide-field Ca^2+^ imaging. (B) A 470 nm excited fluorescence image of WT mouse cortex after multi-site Cal-520 AM loading. Neocortical regions were annotated as frontal (Fro), motor (Mot), somatosensory (Som), and occipital (Occ) cortices.

## Abbreviations

The following abbreviations are used in this manuscript:

WA	Withaferin A
Aβ	Amyloid-β
AD	Alzheimer’s disease
NORT	Novel Object Recognition Test
NLRT	Novel Location Recognition Test
TCST	Three-Chamber Social Test
MWMT	Morris Water Maze Test
ELISA	Enzyme-linked immunosorbent assay
DMSO	Dimethyl sulfoxide
WT	Wild type
2-CAFE	2-Channel Alternating exposure wide-Field Explorer
TBS	Tris-buffered saline
SDS	Sodium dodecyl sulfate
non-REM	Non-rapid eye movement
ROS	Reactive oxygen species
Ctx	Cortex
Hp	Hippocampus
