# Supplementary figures and images for: Withaferin A Rescues Brain Network Dysfunction and Cognitive Deficits in a Mouse Model of Alzheimer’s Disease
Source: Pharmaceuticals (Basel). 2025 May 29;18(6):816. doi: 10.3390/ph18060816 (PMC12196360; doi:10.3390/ph18060816)

A

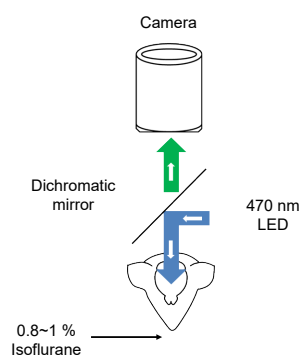

B

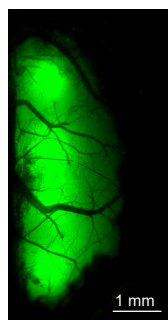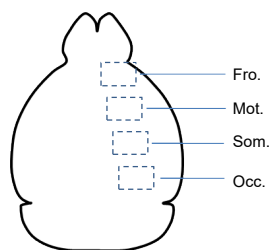

Supplement: Supplementary file 1 [file pharmaceuticals-18-00816-s001.zip › pharmaceuticals-3651916-supplementary.pdf]
